# Supplementary material for: COZOID: contact zone identifier for visual analysis of protein-protein interactions
Source: BMC Bioinformatics. 2018 Apr 6;19:125. doi: 10.1186/s12859-018-2113-6 (PMC5889581; doi:10.1186/s12859-018-2113-6)
Supplement: Supplementary file 2 — Software build. Executable binary file of the software tool. (ZIP 118,784 kb) [file 12859_2018_2113_MOESM2_ESM.zip › cozoid/java/docs/beansbinding-1.2.1-doc.zip!/org/jdesktop/beansbinding/Binding.html]

Binding (Beans Binding)


---


|  |  |  |
| --- | --- | --- |
| **Overview** \| **Package** \| **Class** \| **Use** \| **Tree** \| **Deprecated** \| **Index** \| **Help** | |  |
| **PREV CLASS**   **NEXT CLASS** | **FRAMES**    **NO FRAMES**     **All Classes** |  |
| SUMMARY: NESTED \| FIELD \| CONSTR \| METHOD | DETAIL: FIELD \| CONSTR \| METHOD |  |


---


## org.jdesktop.beansbinding Class Binding<SS,SV,TS,TV>

```
java.lang.Object
  org.jdesktop.beansbinding.Binding<SS,SV,TS,TV>
```

**Type Parameters:**: `SS` - the type of source object: `SV` - the type of value that the source property represents: `TS` - the type of target object: `TV` - the type of value that the target property represents

**Direct Known Subclasses:**: AbstractColumnBinding, AutoBinding

---

``` public abstract class Binding<SS,SV,TS,TV> extends java.lang.Object ```

`Binding` is an abstract class that represents the concept of a
binding between two properties, typically of two objects, and contains
methods for explicitly syncing the values of the two properties. `Binding`
itself does no automatic syncing between property values. Subclasses
will typically keep the values in sync according to some strategy.

Some `Bindings` are managed, often by another `Binding`.
A managed `Binding` does not allow certain methods to be called by
the user. These methods are identified in their documentation.
Subclasses should call `setManaged(true)` to make themselves managed.
`Binding` provides protected versions of the managed methods with the
suffix `"Unmanaged"` for subclasses to use internally without
checking whether or not they are managed.

Any `PropertyResolutionExceptions` thrown by `Property`
objects used by this binding are allowed to flow through to the caller
of the `Binding` methods.

---

| **Nested Class Summary** | |
| --- | --- |
| `static class` | `Binding.SyncFailure`             `SyncFailure` represents a failure to sync (`save` or `refresh`) a `Binding`. |
| `static class` | `Binding.SyncFailureType`             An enumeration representing the reasons a sync (`save` or `refresh`) can fail on a `Binding`. |
| `static class` | `Binding.ValueResult<V>`             Encapsulates the result from calling `getSourceValueForTarget()` or `getTargetValueForSource()`, which can either be a successful value or a failure. |


| **Constructor Summary** | |
| --- | --- |
| `protected` | `Binding(SS sourceObject, Property<SS,SV> sourceProperty, TS targetObject, Property<TS,TV> targetProperty, java.lang.String name)`             Create an instance of `Binding` between two properties of two objects. |


| **Method Summary** | |
| --- | --- |
| `void` | `addBindingListener(BindingListener listener)`             Adds a `BindingListener` to be notified of changes to this `Binding`. |
| `void` | `addPropertyChangeListener(java.beans.PropertyChangeListener listener)`             Adds a `PropertyChangeListener` to be notified when any property of this `Binding` changes. |
| `void` | `addPropertyChangeListener(java.lang.String propertyName, java.beans.PropertyChangeListener listener)`             Adds a `PropertyChangeListener` to be notified when the property identified by the `propertyName` argument changes on this `Binding`. |
| `void` | `bind()`             Binds this binding. |
| `protected abstract  void` | `bindImpl()`             Called by `bind()` to allow subclasses to initiate binding. |
| `protected  void` | `bindUnmanaged()`             A protected version of `bind()` that allows managed subclasses to bind without throwing an exception for being managed. |
| `protected  void` | `firePropertyChange(java.lang.String propertyName, java.lang.Object oldValue, java.lang.Object newValue)`             Sends a `PropertyChangeEvent` to the `PropertyChangeListeners` registered on the `Binding`. |
| `BindingListener[]` | `getBindingListeners()`             Returns the list of `BindingListeners` registered on this `Binding`. |
| `Converter<SV,TV>` | `getConverter()`             Returns the `Binding's Converter`, which may be `null`. |
| `java.lang.String` | `getName()`             Returns the `Binding's` name, which may be `null`. |
| `java.beans.PropertyChangeListener[]` | `getPropertyChangeListeners()`             Returns the list of `PropertyChangeListeners` registered on this `Binding`. |
| `java.beans.PropertyChangeListener[]` | `getPropertyChangeListeners(java.lang.String propertyName)`             Returns the list of `PropertyChangeListeners` registered on this `Binding` for the given property name. |
| `TV` | `getSourceNullValue()`             Returns the value to be returned by `getSourceValueForTarget()` when the source property returns `null` for the source object. |
| `SS` | `getSourceObject()`             Returns the `Binding's` source object, which may be `null`. |
| `Property<SS,SV>` | `getSourceProperty()`             Returns the `Binding's` source property, which may not be `null`. |
| `TV` | `getSourceUnreadableValue()`             If set, returns the value to be returned by `getSourceValueForTarget()` when the source property is unreadable for the source object. |
| `Binding.ValueResult<TV>` | `getSourceValueForTarget()`             Fetches the value of the source property for the source object and returns a `ValueResult` representing that value in terms that can be set on the target property for the target object. |
| `SV` | `getTargetNullValue()`             Returns the value to be returned by `getTargetValueForSource()` when the target property returns `null` for the target object. |
| `TS` | `getTargetObject()`             Returns the `Binding's` target object, which may be `null`. |
| `Property<TS,TV>` | `getTargetProperty()`             Returns the `Binding's` target property, which may not be `null`. |
| `Binding.ValueResult<SV>` | `getTargetValueForSource()`             Fetches the value of the target property for the target object and returns a `ValueResult` representing that value in terms that can be set on the source property for the source object. |
| `Validator<? super SV>` | `getValidator()`             Returns the `Binding's Validator`, which may be `null`. |
| `boolean` | `isBound()`             Returns whether or not this `Binding` is bound. |
| `boolean` | `isManaged()`             Returns whether or not this `Binding` is managed. |
| `boolean` | `isSourceUnreadableValueSet()`             Returns the value of the `sourceUnreadableValueSet` property, which indicates whether or not the `sourceUnreadableValue` property is set on the `Binding`. |
| `protected  void` | `notifySynced()`             Notifies all registered `BindingListeners` of a successful sync (`refresh` or `save`), by calling `synced` on each one. |
| `protected  void` | `notifySyncFailed(Binding.SyncFailure failure)`             Notifies all registered `BindingListeners` of a failure to sync (`refresh` or `save`), by calling `syncFailed` on each one. |
| `protected  java.lang.String` | `paramString()`             Returns a string representing the internal state of the `Binding`. |
| `Binding.SyncFailure` | `refresh()`             Fetches the value of the source property for the source object and sets it as the value of the target property for the target object. |
| `Binding.SyncFailure` | `refreshAndNotify()`             The same as `refresh()` with the additional behavior of notifying all registered `BindingListeners` with `synced` if `refresh` returns `null` or `syncFailed` if `refresh` returns a `SyncFailure`. |
| `protected  Binding.SyncFailure` | `refreshAndNotifyUnmanaged()`             A protected version of `refreshAndNotify()` that allows managed subclasses to refresh and notify without throwing an exception for being managed. |
| `protected  Binding.SyncFailure` | `refreshUnmanaged()`             A protected version of `refresh()` that allows managed subclasses to refresh without throwing an exception for being managed. |
| `void` | `removeBindingListener(BindingListener listener)`             Removes a `BindingListener` from the `Binding`. |
| `void` | `removePropertyChangeListener(java.beans.PropertyChangeListener listener)`             Removes a `PropertyChangeListener` from the `Binding`. |
| `void` | `removePropertyChangeListener(java.lang.String propertyName, java.beans.PropertyChangeListener listener)`             Removes a `PropertyChangeListener` from the `Binding` for the given property name. |
| `Binding.SyncFailure` | `save()`             Fetches the value of the target property for the target object and sets it as the value of the source property for the source object. |
| `Binding.SyncFailure` | `saveAndNotify()`             The same as `save()` with the additional behavior of notifying all registered `BindingListeners` with `synced` if `save` returns `null` or `syncFailed` if `save` returns a `SyncFailure`. |
| `protected  Binding.SyncFailure` | `saveAndNotifyUnmanaged()`             A protected version of `saveAndNotify()` that allows managed subclasses to save and notify without throwing an exception for being managed. |
| `protected  Binding.SyncFailure` | `saveUnmanaged()`             A protected version of `save()` that allows managed subclasses to save without throwing an exception for being managed. |
| `void` | `setConverter(Converter<SV,TV> converter)`             Sets the `Converter` for the `Binding`, which may be `null`. |
| `protected  void` | `setManaged(boolean isManaged)`             Sets whether or not this `Binding` is managed. |
| `void` | `setSourceNullValue(TV sourceNullValue)`             Sets the value to be returned by `getSourceValueForTarget()` when the source property returns `null` for the source object. |
| `void` | `setSourceObject(SS sourceObject)`             Sets the `Binding's` source object, which may be `null`. |
| `protected  void` | `setSourceObjectUnmanaged(SS sourceObject)`             A protected version of `setSourceObject(SS)` that allows managed subclasses to set the source object without throwing an exception for being managed. |
| `protected  void` | `setSourceProperty(Property<SS,SV> sourceProperty)`             Sets the `Binding's` source property. |
| `void` | `setSourceUnreadableValue(TV sourceUnreadableValue)`             Sets the value to be returned by `getSourceValueForTarget()` when the source property is unreadable for the source object. |
| `void` | `setTargetNullValue(SV targetNullValue)`             Sets the value to be returned by `getTargetValueForSource()` when the target property returns `null` for the target object. |
| `void` | `setTargetObject(TS targetObject)`             Sets the `Binding's` target object, which may be `null`. |
| `protected  void` | `setTargetObjectUnmanaged(TS targetObject)`             A protected version of `setTargetObject(TS)` that allows managed subclasses to set the target object without throwing an exception for being managed. |
| `protected  void` | `setTargetProperty(Property<TS,TV> targetProperty)`             Sets the `Binding's` target property. |
| `void` | `setValidator(Validator<? super SV> validator)`             Sets the `Validator` for the `Binding`, which may be `null`. |
| `protected  void` | `sourceChangedImpl(PropertyStateEvent pse)`             Called to indicate that the source property has fired a `PropertyStateEvent` to indicate that its state has changed for the source object. |
| `protected  void` | `targetChangedImpl(PropertyStateEvent pse)`             Called to indicate that the target property has fired a `PropertyStateEvent` to indicate that its state has changed for the target object. |
| `protected  void` | `throwIfBound()`             Throws an IllegalStateException if the `Binding` is bound. |
| `protected  void` | `throwIfManaged()`             Throws an UnsupportedOperationException if the `Binding` is managed. |
| `protected  void` | `throwIfUnbound()`             Throws an IllegalStateException if the `Binding` is unbound. |
| `java.lang.String` | `toString()`             Returns a string representation of the `Binding`. |
| `void` | `unbind()`             Unbinds this binding. |
| `protected abstract  void` | `unbindImpl()`             Called by `unbind()` to allow subclasses to uninitiate binding. |
| `protected  void` | `unbindUnmanaged()`             A protected version of `unbind()` that allows managed subclasses to unbind without throwing an exception for being managed. |
| `void` | `unsetSourceUnreadableValue()`             Unsets the value of the `sourceUnreadableValue` property by clearing the value and setting the value of the `sourceUnreadableValueSet` property to `false`. |

**Methods inherited from class java.lang.Object**

`clone, equals, finalize, getClass, hashCode, notify, notifyAll, wait, wait, wait`

**Constructor Detail**

### Binding

```
protected Binding(SS sourceObject,
                  Property<SS,SV> sourceProperty,
                  TS targetObject,
                  Property<TS,TV> targetProperty,
                  java.lang.String name)
```

:   Create an instance of `Binding` between two properties of two objects.

    **Parameters:**: `sourceObject` - the source object: `sourceProperty` - a property on the source object: `targetObject` - the target object: `targetProperty` - a property on the target object: `name` - a name for the `Binding` **Throws:**: `java.lang.IllegalArgumentException` - if the source property or target property is `null`


**Method Detail**

### setSourceProperty

```
protected final void setSourceProperty(Property<SS,SV> sourceProperty)
```

:   Sets the `Binding's` source property.

    `Binding` fires a property change notification with
    property name `"sourceProperty"` when the value of
    this property changes.

    This method may not be called on a bound binding.

    :   **Parameters:**: `sourceProperty` - the source property **Throws:**: `java.lang.IllegalArgumentException` - if the source property is `null`: `java.lang.IllegalStateException` - if the `Binding` is bound **See Also:**: `isBound()`

---


### setTargetProperty

```
protected final void setTargetProperty(Property<TS,TV> targetProperty)
```

:   Sets the `Binding's` target property.

    `Binding` fires a property change notification with
    property name `"targetProperty"` when the value of
    this property changes.

    This method may not be called on a bound binding.

    :   **Parameters:**: `targetProperty` - the target property **Throws:**: `java.lang.IllegalArgumentException` - if the target property is `null`: `java.lang.IllegalStateException` - if the `Binding` is bound **See Also:**: `isBound()`

---


### getName

```
public final java.lang.String getName()
```

:   Returns the `Binding's` name, which may be `null`.

    :   **Returns:**: the `Binding's` name, or `null`

---


### getSourceProperty

```
public final Property<SS,SV> getSourceProperty()
```

:   Returns the `Binding's` source property, which may not be `null`.

    :   **Returns:**: the `Binding's` source property, `non-null` **See Also:**: `setSourceProperty(org.jdesktop.beansbinding.Property)`

---


### getTargetProperty

```
public final Property<TS,TV> getTargetProperty()
```

:   Returns the `Binding's` target property, which may not be `null`.

    :   **Returns:**: the `Binding's` target property, `non-null` **See Also:**: `setTargetProperty(org.jdesktop.beansbinding.Property)`

---


### getSourceObject

```
public final SS getSourceObject()
```

:   Returns the `Binding's` source object, which may be `null`.

    :   **Returns:**: the `Binding's` source object, or `null` **See Also:**: `setSourceObject(SS)`

---


### getTargetObject

```
public final TS getTargetObject()
```

:   Returns the `Binding's` target object, which may be `null`.

    :   **Returns:**: the `Binding's` target object, or `null` **See Also:**: `setTargetObject(TS)`

---


### setSourceObject

```
public final void setSourceObject(SS sourceObject)
```

:   Sets the `Binding's` source object, which may be `null`.

    `Binding` fires a property change notification with
    property name `"sourceObject"` when the value of
    this property changes.

    This method may not be called on a managed or bound binding.

    :   **Parameters:**: `sourceObject` - the source object, or `null` **Throws:**: `java.lang.UnsupportedOperationException` - if the `Binding` is managed: `java.lang.IllegalStateException` - if the `Binding` is bound **See Also:**: `isManaged()`, `isBound()`

---


### setSourceObjectUnmanaged

```
protected final void setSourceObjectUnmanaged(SS sourceObject)
```

:   A protected version of `setSourceObject(SS)` that allows managed
    subclasses to set the source object without throwing an exception
    for being managed.

    :   **Parameters:**: `sourceObject` - the source object, or `null` **Throws:**: `java.lang.IllegalStateException` - if the `Binding` is bound **See Also:**: `isManaged()`, `isBound()`

---


### setTargetObject

```
public final void setTargetObject(TS targetObject)
```

:   Sets the `Binding's` target object, which may be `null`.

    `Binding` fires a property change notification with
    property name `"targetObject"` when the value of
    this property changes.

    This method may not be called on a managed or bound binding.

    :   **Parameters:**: `targetObject` - the target object, or `null` **Throws:**: `java.lang.UnsupportedOperationException` - if the `Binding` is managed: `java.lang.IllegalStateException` - if the `Binding` is bound **See Also:**: `isManaged()`, `isBound()`

---


### setTargetObjectUnmanaged

```
protected final void setTargetObjectUnmanaged(TS targetObject)
```

:   A protected version of `setTargetObject(TS)` that allows managed
    subclasses to set the target object without throwing an exception
    for being managed.

    :   **Parameters:**: `targetObject` - the target object, or `null` **Throws:**: `java.lang.IllegalStateException` - if the `Binding` is bound **See Also:**: `isManaged()`, `isBound()`

---


### setValidator

```
public final void setValidator(Validator<? super SV> validator)
```

:   Sets the `Validator` for the `Binding`, which may be `null`.

    `Binding` fires a property change notification with
    property name `"validator"` when the value of
    this property changes.

    This method may not be called on a bound binding.

    See the documentation on `getTargetValueForSource()` for details on how
    a `Binding's Validator` is used.

    :   **Parameters:**: `validator` - the `Validator`, or `null` **Throws:**: `java.lang.IllegalStateException` - if the `Binding` is bound **See Also:**: `isBound()`

---


### getValidator

```
public final Validator<? super SV> getValidator()
```

:   Returns the `Binding's Validator`, which may be `null`.

    :   **Returns:**: the `Binding's Validator`, or `null` **See Also:**: `setValidator(org.jdesktop.beansbinding.Validator super SV)`

---


### setConverter

```
public final void setConverter(Converter<SV,TV> converter)
```

:   Sets the `Converter` for the `Binding`, which may be `null`.

    `Binding` fires a property change notification with
    property name `"converter"` when the value of
    this property changes.

    This method may not be called on a bound binding.

    See the documentation on `getTargetValueForSource()` and
    `getSourceValueForTarget()` for details on how
    a `Binding's Converter` is used.

    :   **Parameters:**: `converter` - the `Converter`, or `null` **Throws:**: `java.lang.IllegalStateException` - if the `Binding` is bound **See Also:**: `isBound()`

---


### getConverter

```
public final Converter<SV,TV> getConverter()
```

:   Returns the `Binding's Converter`, which may be `null`.

    :   **Returns:**: the `Binding's Converter`, or `null` **See Also:**: `setConverter(org.jdesktop.beansbinding.Converter)`

---


### setSourceNullValue

```
public final void setSourceNullValue(TV sourceNullValue)
```

:   Sets the value to be returned by `getSourceValueForTarget()`
    when the source property returns `null` for the source object.
    The default for this property is `null`.

    `Binding` fires a property change notification with
    property name `"sourceNullValue"` when the value of
    this property changes.

    This method may not be called on a bound binding.

    :   **Parameters:**: `sourceNullValue` - the value, or `null` **Throws:**: `java.lang.IllegalStateException` - if the `Binding` is bound

---


### getSourceNullValue

```
public final TV getSourceNullValue()
```

:   Returns the value to be returned by `getSourceValueForTarget()`
    when the source property returns `null` for the source object.
    The default for this property is `null`.

    :   **Returns:**: the value that replaces a source value of `null`, or `null` if there is no replacement **See Also:**: `setSourceNullValue(TV)`

---


### setTargetNullValue

```
public final void setTargetNullValue(SV targetNullValue)
```

:   Sets the value to be returned by `getTargetValueForSource()`
    when the target property returns `null` for the target object.
    The default for this property is `null`.

    `Binding` fires a property change notification with
    property name `"targetNullValue"` when the value of
    this property changes.

    This method may not be called on a bound binding.

    :   **Parameters:**: `targetNullValue` - the value, or `null` **Throws:**: `java.lang.IllegalStateException` - if the `Binding` is bound

---


### getTargetNullValue

```
public final SV getTargetNullValue()
```

:   Returns the value to be returned by `getTargetValueForSource()`
    when the target property returns `null` for the target object.
    The default for this property is `null`.

    :   **Returns:**: the value that replaces a target value of `null`, or `null` if there is no replacement **See Also:**: `setTargetNullValue(SV)`

---


### setSourceUnreadableValue

```
public final void setSourceUnreadableValue(TV sourceUnreadableValue)
```

:   Sets the value to be returned by `getSourceValueForTarget()`
    when the source property is unreadable for the source object.
    Calling this method stores the given value and indicates that
    `getSourceValueForTarget` should use it, by setting the
    `sourceUnreadableValueSet` property to `true`.

    By default, the `sourceUnreadableValue` property is unset,
    indicated by the `sourceUnreadableValueSet` property being
    `false`.

    Setting this property to `null` acts the same as setting it to
    any other value. To return the property to the unset state (clearing
    the value and setting `sourceUnreadableValueSet` back to
    `false`) call `unsetSourceUnreadableValue()`.

    If this property was previously unset, this method fires a property
    change notification with property name `"sourceUnreadableValueSet"`.
    For all invocations, it also fires a property change notification with
    property name `"sourceUnreadableValue"`, if necessary, to indicate
    a change in the property value. If previously unset, the event will
    indicate an old value of `null`.

    This method may not be called on a bound binding.

    :   **Parameters:**: `sourceUnreadableValue` - the value, which may be `null` **Throws:**: `java.lang.IllegalStateException` - if the `Binding` is bound **See Also:**: `isSourceUnreadableValueSet()`, `getSourceUnreadableValue()`

---


### unsetSourceUnreadableValue

```
public final void unsetSourceUnreadableValue()
```

:   Unsets the value of the `sourceUnreadableValue` property by clearing
    the value and setting the value of the `sourceUnreadableValueSet`
    property to `false`.

    If the property was previously set, fires a property change notification
    with property name `"sourceUnreadableValueSet"`, and a property
    change notification with property name `"sourceUnreadableValue"`.
    The event for the latter notification will have a new value of `null`.

    See the documentation for `setSourceUnreadableValue(TV)` for more
    information on the `sourceUnreadableValue` property.

    This method may not be called on a bound binding.

    :   **Throws:**: `java.lang.IllegalStateException` - if the `Binding` is bound **See Also:**: `isSourceUnreadableValueSet()`, `getSourceUnreadableValue()`

---


### isSourceUnreadableValueSet

```
public final boolean isSourceUnreadableValueSet()
```

:   Returns the value of the `sourceUnreadableValueSet` property,
    which indicates whether or not the `sourceUnreadableValue` property
    is set on the `Binding`.

    See the documentation for `setSourceUnreadableValue(TV)` for more
    information on the `sourceUnreadableValue` property.

    :   **Returns:**: whether or not the `sourceUnreadableValue` property is set on the `Binding` **See Also:**: `unsetSourceUnreadableValue()`, `getSourceUnreadableValue()`

---


### getSourceUnreadableValue

```
public final TV getSourceUnreadableValue()
```

:   If set, returns the value to be returned by `getSourceValueForTarget()`
    when the source property is unreadable for the source object. Throws
    `UnsupportedOperationException` if the property is not set,
    as indicated by `isSourceUnreadableValueSet()`.

    See the documentation for `setSourceUnreadableValue(TV)` for more
    information on this property.

    :   **Returns:**: the value that replaces an unreadable source value, which may be `null` **Throws:**: `java.lang.UnsupportedOperationException` - if the property is not set, as indicated by `isSourceUnreadableValueSet` **See Also:**: `unsetSourceUnreadableValue()`

---


### addBindingListener

```
public final void addBindingListener(BindingListener listener)
```

:   Adds a `BindingListener` to be notified of changes to this `Binding`.
    Does nothing if the listener is `null`. If a listener is added more than once,
    notifications are sent to that listener once for every time that it has
    been added. The ordering of listener notification is unspecified.

    :   **Parameters:**: `listener` - the listener to add

---


### removeBindingListener

```
public final void removeBindingListener(BindingListener listener)
```

:   Removes a `BindingListener` from the `Binding`. Does
    nothing if the listener is `null` or is not one of those registered.
    If the listener being removed was registered more than once, only one
    occurrence of the listener is removed from the list of listeners.
    The ordering of listener notification is unspecified.

    :   **Parameters:**: `listener` - the listener to remove **See Also:**: `addBindingListener(org.jdesktop.beansbinding.BindingListener)`

---


### getBindingListeners

```
public final BindingListener[] getBindingListeners()
```

:   Returns the list of `BindingListeners` registered on this
    `Binding`. Order is undefined. Returns an empty array if there are
    no listeners.

    :   **Returns:**: the list of `BindingListeners` registered on this `Binding` **See Also:**: `addBindingListener(org.jdesktop.beansbinding.BindingListener)`

---


### getSourceValueForTarget

```
public final Binding.ValueResult<TV> getSourceValueForTarget()
```

:   Fetches the value of the source property for the source object and
    returns a `ValueResult` representing that value in terms that
    can be set on the target property for the target object.

    First, if the target property is not writeable for the target object,
    a `ValueResult` is returned representing a failure
    with failure type `SyncFailureType.TARGET_UNWRITEABLE`.
    Then, if the source property is unreadable for the source object,
    the value of `isSourceUnreadableValueSet()` is checked. If `true`
    then a `ValueResult` is returned containing the value of the
    `Binding's` `getSourceUnreadableValue()`. Otherwise a
    `ValueResult` is returned representing a failure with failure
    type `SyncFailureType.SOURCE_UNREADABLE`.

    Next, the value of the source property is fetched for the source
    object. If the value is `null`, a `ValueResult` is
    returned containing the value of the `Binding's`
    `getSourceNullValue()`. If the value is `non-null`,
    the `Binding's Converter`, if any, is run to convert
    the value from source type to the target property's
    `getWriteType`, by calling its `convertForward`
    method with the value. If no `Converter` is registered,
    a set of default converters is checked to see if one of them
    can convert the value to the target type. Finally, the value
    (converted or not) is cast to the target write type.

    This final value is returned in a `ValueResult`.

    Any `RuntimeException` or `ClassCastException` thrown by a
    converter or the final cast is propogated up to the caller of this method.

    :   **Returns:**: a `ValueResult` as described above **Throws:**: `java.lang.RuntimeException` - if thrown by any of the converters: `java.lang.ClassCastException` - if thrown by a converter or the final cast

---


### getTargetValueForSource

```
public final Binding.ValueResult<SV> getTargetValueForSource()
```

:   Fetches the value of the target property for the target object and
    returns a `ValueResult` representing that value in terms that
    can be set on the source property for the source object.

    First, if the source property is not writeable for the source object,
    a `ValueResult` is returned representing a failure
    with failure type `SyncFailureType.SOURCE_UNWRITEABLE`.
    Then, if the target property is not readable for the target object,
    a `ValueResult` is returned representing a failure
    with failure type `SyncFailureType.TARGET_UNREADABLE`.

    Next, the value of the target property is fetched for the target
    object. If the value is `null`, a `ValueResult` is
    returned containing the value of the `Binding's`
    `getTargetNullValue()`. If the value is `non-null`,
    the `Binding's Converter`, if any, is run to convert
    the value from target type to the source property's
    `getWriteType`, by calling its `convertReverse`
    method with the value. If no `Converter` is registered,
    a set of default converters is checked to see if one of them
    can convert the value to the source type. Finally, the value
    (converted or not) is cast to the source write type.

    If a converter throws a `RuntimeException` other than
    `ClassCastException`, this method returns a
    `ValueResult` containing the failure, with failure type
    `SyncFailureType.CONVERSION_FAILURE`.

    As the last step, the `Binding's Validator`, if any, is called
    upon to validate the final value. If the `Validator`
    returns `non-null` from its `validate` method,
    a `ValueResult` is returned containing the validation
    result, with failure type `SyncFailureType.VALIDATION_FAILURE`.
    Otherwise a `ValueResult` is returned containing the
    final validated value.

    Any `ClassCastException` thrown by a converter or the final
    cast is propogated up to the caller of this method.

    :   **Returns:**: a `ValueResult` as described above **Throws:**: `java.lang.ClassCastException` - if thrown by a converter or the final cast

---


### bind

```
public final void bind()
```

:   Binds this binding. Calls `bindImpl()` to allow subclasses
    to initiate binding, adds a `PropertyStateListener` to the source
    property for the source object and the target property for the target
    object to start tracking changes, notifies all registered
    `BindingListeners` that the binding has become bound, and
    fires a property change notification to indicate a change to the
    `"bound"` property.

    :   **Throws:**: `java.lang.UnsupportedOperationException` - if the `Binding` is managed: `java.lang.IllegalStateException` - if the `Binding` is already bound **See Also:**: `isBound()`, `isManaged()`, `unbind()`

---


### bindUnmanaged

```
protected final void bindUnmanaged()
```

:   A protected version of `bind()` that allows managed
    subclasses to bind without throwing an exception
    for being managed.

    :   **Throws:**: `java.lang.IllegalStateException` - if the `Binding` is bound **See Also:**: `isManaged()`, `isBound()`

---


### bindImpl

```
protected abstract void bindImpl()
```

:   Called by `bind()` to allow subclasses to initiate binding.
    Subclasses typically need not install `PropertyStateListeners`
    on the source property and target property as they will be notified
    by calls to `sourceChangedImpl(org.jdesktop.beansbinding.PropertyStateEvent)` and `targetChangedImpl(org.jdesktop.beansbinding.PropertyStateEvent)`
    when the source and target properties change respectively.

    :   **See Also:**: `unbindImpl()`

---


### unbind

```
public final void unbind()
```

:   Unbinds this binding. Removes the `PropertyStateListeners`
    added by `bind`, calls `unbindImpl()` to allow subclasses
    to uninitiate binding, notifies all registered `BindingListeners`
    that the binding has become unbound, and fires a property change
    notification to indicate a change to the `"bound"` property.

    :   **Throws:**: `java.lang.UnsupportedOperationException` - if the `Binding` is managed: `java.lang.IllegalStateException` - if the `Binding` is not bound **See Also:**: `isBound()`, `isManaged()`, `bind()`

---


### unbindUnmanaged

```
protected final void unbindUnmanaged()
```

:   A protected version of `unbind()` that allows managed
    subclasses to unbind without throwing an exception
    for being managed.

    :   **Throws:**: `java.lang.IllegalStateException` - if the `Binding` is not bound **See Also:**: `isManaged()`, `isBound()`

---


### unbindImpl

```
protected abstract void unbindImpl()
```

:   Called by `unbind()` to allow subclasses to uninitiate binding.

    :   **See Also:**: `bindImpl()`

---


### isBound

```
public final boolean isBound()
```

:   Returns whether or not this `Binding` is bound.

    `Binding` fires a property change notification with
    property name `"bound"` when the value of
    this property changes.

    :   **Returns:**: whether or not the `Binding` is bound **See Also:**: `bind()`, `unbind()`

---


### setManaged

```
protected final void setManaged(boolean isManaged)
```

:   Sets whether or not this `Binding` is managed. Some
    `Bindings` are managed, often by another `Binding`.
    A managed `Binding` does not allow certain methods to be called by
    the user. These methods are identified in their documentation.
    Subclasses should call `setManaged(true)` to make themselves managed.
    `Binding` provides protected versions of the managed methods, with the
    suffix `"Unmanaged"`, for subclasses to use internally without
    checking whether or not they are managed.

---


### isManaged

```
public final boolean isManaged()
```

:   Returns whether or not this `Binding` is managed. Some
    `Bindings` are managed, often by another `Binding`.
    A managed `Binding` does not allow certain methods to be called by
    the user. These methods are identified in their documentation.
    Subclasses should call `setManaged(true)` to make themselves managed.
    `Binding` provides protected versions of the managed methods, with the
    suffix `"Unmanaged"`, for subclasses to use internally without
    checking whether or not they are managed.

    :   **Returns:**: whether or not the `Binding` is managed **See Also:**: `setManaged(boolean)`

---


### notifySynced

```
protected final void notifySynced()
```

:   Notifies all registered `BindingListeners` of a successful
    sync (`refresh` or `save`), by calling `synced`
    on each one.

---


### notifySyncFailed

```
protected final void notifySyncFailed(Binding.SyncFailure failure)
```

:   Notifies all registered `BindingListeners` of a failure to
    sync (`refresh` or `save`), by calling
    `syncFailed` on each one.

    :   **Parameters:**: `failure` - the reason that the sync failed

---


### refreshAndNotify

```
public final Binding.SyncFailure refreshAndNotify()
```

:   The same as `refresh()` with the additional
    behavior of notifying all registered `BindingListeners`
    with `synced` if `refresh` returns `null`
    or `syncFailed` if `refresh` returns a
    `SyncFailure`.

    :   **Returns:**: the return value from the call to `refresh` **Throws:**: `java.lang.UnsupportedOperationException` - if the `Binding` is managed: `java.lang.RuntimeException` - as specified by `refresh()`: `java.lang.ClassCastException` - as specified by `refresh()` **See Also:**: `isManaged()`

---


### refreshAndNotifyUnmanaged

```
protected final Binding.SyncFailure refreshAndNotifyUnmanaged()
```

:   A protected version of `refreshAndNotify()` that allows managed
    subclasses to refresh and notify without throwing an exception
    for being managed.

    :   **Returns:**: the return value from the call to `refresh` **Throws:**: `java.lang.RuntimeException` - as specified by `refresh()`: `java.lang.ClassCastException` - as specified by `refresh()` **See Also:**: `isManaged()`

---


### saveAndNotify

```
public final Binding.SyncFailure saveAndNotify()
```

:   The same as `save()` with the additional
    behavior of notifying all registered `BindingListeners`
    with `synced` if `save` returns `null`
    or `syncFailed` if `save` returns a
    `SyncFailure`.

    :   **Returns:**: the return value from the call to `save` **Throws:**: `java.lang.UnsupportedOperationException` - if the `Binding` is managed: `java.lang.ClassCastException` - as specified by `refresh()` **See Also:**: `isManaged()`

---


### saveAndNotifyUnmanaged

```
protected final Binding.SyncFailure saveAndNotifyUnmanaged()
```

:   A protected version of `saveAndNotify()` that allows managed
    subclasses to save and notify without throwing an exception
    for being managed.

    :   **Returns:**: the return value from the call to `save` **Throws:**: `java.lang.ClassCastException` - as specified by `save()` **See Also:**: `isManaged()`

---


### refresh

```
public final Binding.SyncFailure refresh()
```

:   Fetches the value of the source property for the source object and sets
    it as the value of the target property for the target object.
    First calls `getSourceValueForTarget()`. If the return value
    from that method represents a failure, this method returns the failure.
    Otherwise, it calls `setValue` on the target property for the
    target object with the value obtained from the source.

    :   **Returns:**: the reason for failure if the binding could not be refreshed, or `null` for success **Throws:**: `java.lang.UnsupportedOperationException` - if the `Binding` is managed: `java.lang.RuntimeException` - if thrown by `getSourceValueForTarget()`: `java.lang.ClassCastException` - if thrown by `getSourceValueForTarget()` **See Also:**: `isManaged()`, `save()`

---


### refreshUnmanaged

```
protected final Binding.SyncFailure refreshUnmanaged()
```

:   A protected version of `refresh()` that allows managed
    subclasses to refresh without throwing an exception
    for being managed.

    :   **Returns:**: the reason for failure if the binding could not be refreshed, or `null` for success **Throws:**: `java.lang.RuntimeException` - if thrown by `getSourceValueForTarget()`: `java.lang.ClassCastException` - if thrown by `getSourceValueForTarget()` **See Also:**: `isManaged()`

---


### save

```
public final Binding.SyncFailure save()
```

:   Fetches the value of the target property for the target object and sets
    it as the value of the source property for the source object.
    First calls `getTargetValueForSource()`. If the return value
    from that method represents a failure, this method returns the failure.
    Otherwise, it calls `setValue` on the source property for the
    source object with the value obtained from the target.

    :   **Returns:**: the reason for failure if the binding could not be saved, or `null` for success **Throws:**: `java.lang.UnsupportedOperationException` - if the `Binding` is managed: `java.lang.ClassCastException` - if thrown by `getTargetValueForSource()` **See Also:**: `isManaged()`, `refresh()`

---


### saveUnmanaged

```
protected final Binding.SyncFailure saveUnmanaged()
```

:   A protected version of `save()` that allows managed
    subclasses to save without throwing an exception
    for being managed.

    :   **Returns:**: the reason for failure if the binding could not be saved, or `null` for success **Throws:**: `java.lang.ClassCastException` - if thrown by `getTargetValueForSource()` **See Also:**: `isManaged()`

---


### throwIfManaged

```
protected final void throwIfManaged()
```

:   Throws an UnsupportedOperationException if the `Binding` is managed.
    Useful for calling at the beginning of method implementations that
    shouldn't be called on managed `Bindings`

    :   **Throws:**: `java.lang.UnsupportedOperationException` - if the `Binding` is managed **See Also:**: `isManaged()`

---


### throwIfBound

```
protected final void throwIfBound()
```

:   Throws an IllegalStateException if the `Binding` is bound.
    Useful for calling at the beginning of method implementations that
    shouldn't be called when the `Binding` is bound.

    :   **Throws:**: `java.lang.IllegalStateException` - if the `Binding` is bound.

---


### throwIfUnbound

```
protected final void throwIfUnbound()
```

:   Throws an IllegalStateException if the `Binding` is unbound.
    Useful for calling at the beginning of method implementations that should
    only be called when the `Binding` is bound.

    :   **Throws:**: `java.lang.IllegalStateException` - if the `Binding` is unbound.

---


### toString

```
public java.lang.String toString()
```

:   Returns a string representation of the `Binding`. This
    method is intended to be used for debugging purposes only, and
    the content and format of the returned string may vary between
    implementations. The returned string may be empty but may not
    be `null`.

    :   **Overrides:**: `toString` in class `java.lang.Object`
    :   **Returns:**: a string representation of this `Binding`

---


### paramString

```
protected java.lang.String paramString()
```

:   Returns a string representing the internal state of the `Binding`.
    This method is intended to be used for debugging purposes only,
    and the content and format of the returned string may vary between
    implementations. The returned string may be empty but may not
    be `null`.

    :   **Returns:**: a string representing the state of the `Binding`.

---


### sourceChangedImpl

```
protected void sourceChangedImpl(PropertyStateEvent pse)
```

:   Called to indicate that the source property has fired a
    `PropertyStateEvent` to indicate that its state has changed for
    the source object. Called after the `Binding` has notified
    any property change listeners and `BindingListeners` that
    the source value has been edited (only if the `PropertyStateEvent`
    represents a value change). This method is useful for subclasses
    to detect source changes and perform syncing as appropriate.

---


### targetChangedImpl

```
protected void targetChangedImpl(PropertyStateEvent pse)
```

:   Called to indicate that the target property has fired a
    `PropertyStateEvent` to indicate that its state has changed for
    the target object. Called after the `Binding` has notified
    any property change listeners and `BindingListeners` that
    the target value has been edited (only if the `PropertyStateEvent`
    represents a value change). This method is useful for subclasses
    to detect target changes and perform syncing as appropriate.

---


### addPropertyChangeListener

```
public final void addPropertyChangeListener(java.beans.PropertyChangeListener listener)
```

:   Adds a `PropertyChangeListener` to be notified when any property of
    this `Binding` changes. Does nothing if the listener is
    `null`. If a listener is added more than once, notifications are
    sent to that listener once for every time that it has been added.
    The ordering of listener notification is unspecified.

    `Binding` fires property change notification for the following
    properties:

    - `sourceProperty`- `targetProperty`- `sourceObject`- `targetObject`- `validator`- `converter`- `sourceNullValue`- `targetNullValue`- `sourceUnreadableValueSet`- `sourceUnreadableValue`- `bound`

    For other types of `Binding` notifications register a
    `BindingListener`.

    :   **Parameters:**: `listener` - the listener to add **See Also:**: `addBindingListener(org.jdesktop.beansbinding.BindingListener)`

---


### addPropertyChangeListener

```
public final void addPropertyChangeListener(java.lang.String propertyName,
                                            java.beans.PropertyChangeListener listener)
```

:   Adds a `PropertyChangeListener` to be notified when the property identified
    by the `propertyName` argument changes on this `Binding`.
    Does nothing if the property name or listener is `null`.
    If a listener is added more than once, notifications are
    sent to that listener once for every time that it has been added.
    The ordering of listener notification is unspecified.

    `Binding` fires property change notification for the following
    properties:

    - `sourceProperty`- `targetProperty`- `sourceObject`- `targetObject`- `validator`- `converter`- `sourceNullValue`- `targetNullValue`- `sourceUnreadableValueSet`- `sourceUnreadableValue`- `bound`

    For other types of `Binding` notifications register a
    `BindingListener`.

    :   **Parameters:**: `propertyName` - the name of the property to listen for changes on: `listener` - the listener to add

---


### removePropertyChangeListener

```
public final void removePropertyChangeListener(java.beans.PropertyChangeListener listener)
```

:   Removes a `PropertyChangeListener` from the `Binding`. Does
    nothing if the listener is `null` or is not one of those registered.
    If the listener being removed was registered more than once, only one
    occurrence of the listener is removed from the list of listeners.
    The ordering of listener notification is unspecified.

    :   **Parameters:**: `listener` - the listener to remove **See Also:**: `addPropertyChangeListener(java.beans.PropertyChangeListener)`

---


### removePropertyChangeListener

```
public final void removePropertyChangeListener(java.lang.String propertyName,
                                               java.beans.PropertyChangeListener listener)
```

:   Removes a `PropertyChangeListener` from the `Binding` for the given
    property name. Does nothing if the property name or listener is
    `null` or the listener is not one of those registered.
    If the listener being removed was registered more than once, only one
    occurrence of the listener is removed from the list of listeners.
    The ordering of listener notification is unspecified.

    :   **Parameters:**: `propertyName` - the name of the property to remove the listener for: `listener` - the listener to remove **See Also:**: `addPropertyChangeListener(String, PropertyChangeListener)`

---


### getPropertyChangeListeners

```
public final java.beans.PropertyChangeListener[] getPropertyChangeListeners()
```

:   Returns the list of `PropertyChangeListeners` registered on this
    `Binding`. Order is undefined. Returns an empty array if there are
    no listeners.

    :   **Returns:**: the list of `PropertyChangeListeners` registered on this `Binding` **See Also:**: `addPropertyChangeListener(java.beans.PropertyChangeListener)`

---


### getPropertyChangeListeners

```
public final java.beans.PropertyChangeListener[] getPropertyChangeListeners(java.lang.String propertyName)
```

:   Returns the list of `PropertyChangeListeners` registered on this
    `Binding` for the given property name. Order is undefined. Returns an empty array
    if there are no listeners registered for the property name.

    :   **Parameters:**: `propertyName` - the property name to retrieve the listeners for **Returns:**: the list of `PropertyChangeListeners` registered on this `Binding` for the given property name **See Also:**: `addPropertyChangeListener(String, PropertyChangeListener)`

---


### firePropertyChange

```
protected final void firePropertyChange(java.lang.String propertyName,
                                        java.lang.Object oldValue,
                                        java.lang.Object newValue)
```

:   Sends a `PropertyChangeEvent` to the `PropertyChangeListeners`
    registered on the `Binding`.

    :   **Parameters:**: `propertyName` - the name of the property that's changed: `oldValue` - the old value of the property: `newValue` - the new value of the property


---


|  |  |  |
| --- | --- | --- |
| **Overview** \| **Package** \| **Class** \| **Use** \| **Tree** \| **Deprecated** \| **Index** \| **Help** | |  |
| **PREV CLASS**   **NEXT CLASS** | **FRAMES**    **NO FRAMES**     **All Classes** |  |
| SUMMARY: NESTED \| FIELD \| CONSTR \| METHOD | DETAIL: FIELD \| CONSTR \| METHOD |  |


---
